# Neurodegenerative brain changes are associated with area deprivation in the United Kingdom: findings from the Brains for Dementia Research study

**DOI:** 10.1186/s40478-021-01301-8

**Published:** 2021-12-19

**Authors:** Calum A. Hamilton, Fiona E. Matthews, Daniel Erskine, Johannes Attems, Alan J. Thomas

**Affiliations:** 1 Translational and Clinical Research Institute, Newcastle University, Newcastle upon Tyne, England; 2 Population Health Sciences Institute, Newcastle University, Newcastle upon Tyne, England

**Keywords:** Social disadvantage, Neuropathology, Alzheimer’s disease

## Abstract

Socioeconomic disadvantage is associated with greater risk of dementia. This has been theorised to reflect inequalities in cognitive reserve, healthcare access, lifestyle, and other health factors which may contribute to the clinical manifestation of dementia. We aimed to assess whether area deprivation in the United Kingdom was associated with greater risk or severity of the specific neurodegenerative diseases which lead to dementia in a multi-centre cohort with autopsy assessment. Participants underwent clinical assessment prior to brain tissue donation post-mortem. Each then underwent detailed, standardised neuropathological assessment. National area deprivation statistics were derived for each participant’s neighbourhood, for use as a predictor in binary and ordinal logistic models assessing the respective presence and severity of staging of key neuropathological changes, adjusting for theorised confounders. Individuals from among the 20% most deprived neighbourhoods in the United Kingdom had significantly higher neurofibrillary tangle and neuritic plaque staging, and increased risk of cerebral amyloid angiopathy. These findings were not explained by a greater risk of diabetes or hypertension, APOE genotype, alcohol misuse or tobacco smoking, sex, or age differences. A sensitivity analysis conditioning on baseline cognitive impairment did not meaningfully change the observed association. Socioeconomic disadvantage may contribute to dementia incidence through a greater severity of specific neuropathological changes (neurofibrillary tangles, neuritic plaques, and cerebral amyloid angiopathy), independent of other indirect influences. Mechanisms through which deprivation is associated with these require further exploration.

**Supplementary Information:**

The online version contains supplementary material available at 10.1186/s40478-021-01301-8.

## Introduction

Dementia is responsible for considerable financial, health, and quality of life costs to individuals with this condition, caregivers, and healthcare services. With ageing populations and no disease-modifying therapies in sight, prevalence and costs are expected to rise globally over the coming decades [1]. There is a growing inequality in dementia incidence between lower-middle income countries, which are seeing greater increases in dementia incidence, and high-income countries [2].

In addition to global inequalities, socioeconomic disadvantage may also be responsible for intranational inequalities in incidence, care and outcomes of dementia. Greater disadvantage may be associated with lower cognitive reserve [3], leading to a lower pathological threshold for clinically manifest dementia [4], and lower-than-expected cognitive function relative to the severity of brain disease [5]. In the United Kingdom, greater local area deprivation is also associated with poorer quality of life after diagnosis of dementia [6], and reduced access to dementia treatments [7].

It has been theorised [2] that addressing several deprivation-associated modifiable risk factors could serve to reduce dementia inequalities not only through increasing cognitive reserve, but also by attenuating the neuropathological changes responsible for dementia through bettering overall brain health: specifically, reducing incidence of cerebrovascular disease (CVD); one of a number of contributors to dementia, including common neurodegenerative diseases such as Alzheimer’s disease (AD) and Lewy body (LB) disease. However, that socioeconomic disadvantage and associated health factors directly contribute to the risk or severity of neuropathological changes, rather than risk of dementia diagnosis, has not been clearly demonstrated.

Recent research has suggested that, in the United States, increased neighbourhood deprivation may be associated with increased risks of meeting neuropathological criteria for AD [8], suggesting that there may be deprivation-related inequalities in non-CVD neuropathological change. However, it is unclear which specific pathological changes (i.e., tau or amyloid) drive this effect in AD, whether this is associated with other common dementia-associated brain diseases (e.g. LB disease or CVD), and whether this effect is mediated by recognised dementia-related risk factors (e.g. APOE ε4 status, diabetes, or smoking).

As of 2021, the Brains for Dementia Research (BDR) initiative, including brain banks from a variety of regions across England and Wales, holds brain tissue from over 900 donors [9]. This includes those both with and without dementia. The majority of donors have provided clinical assessment(s) and detailed medical history prior to death, before undergoing detailed neuropathological assessment, and many have undergone APOE genotyping. Using this resource, we aimed to assess if those residing in more deprived areas of the United Kingdom were at risk of greater incidence or severity of specific neurodegenerative or cerebrovascular changes, while controlling for other important covariates such as APOE status and cerebrovascular risk factors.

We hypothesised that higher area deprivation would be associated with greater severity of staged neuropathological changes (hierarchical distribution and severity of amyloid plaques and neurofibrillary tangles characteristic of AD, and distribution of LBs), and greater incidence of binarised findings (LB disease of any region, subcortical infarcts, cerebral amyloid angiopathy, white matter arteriosclerosis, and TDP-43).

## Materials and methods

### Participants

As previously described [9], participants were brain tissue donors recruited at six sites across the United Kingdom (Bristol, London, Cardiff, Manchester, Newcastle and Oxford) through public research involvement events, support groups, charity newsletters, online publicity, memory clinics, or through their involvement in other clinical research. This was intended to provide a broad and representative sample by reducing the influence of samples from a single source.

Brain tissue donors provided written, informed consent, and donations were conducted with the agreement of a consultee or family member, and facilitated by a nominated representative [9]. Eight-hundred and forty-six cases were available in the BDR cohort who had 1) completed at least one antemortem BDR clinical assessment and 2) undergone detailed neuropathological assessment at the time of data locking. This number excluded those who died before the age of 60.

### Design and procedure

#### Clinical assessment

Prior to donation, participants underwent one or more clinical assessment(s), repeated approximately annually with a research nurse or psychologist. These clinical assessments provided information on individual demographics, medical history, APOE genotype, as well as other measures not included here. Reported history of hypertension, diabetes mellitus, smoking, and heavy alcohol use were derived from the Cambridge Examination for Mental Disorders of the Elderly – Medical History (CAMDEX), or from medical notes.

Clinical dementia rating (CDR) was completed by a trained psychologist or research nurse based on face-to-face assessment.

#### Neuropathological assessment

Antibodies used were AT8 for tau, 4G8 for beta-amyloid, and KM51 for alpha-synuclein. Standardised neuropathological assessments were conducted at each site, as described previously[9, 10]: Thal phasing of amyloid-beta deposition [11], Braak staging of neurofibrillary tangle presence [12], and CERAD scoring of neuritic plaque accumulation[13] were rated in a semi-quantitative manner by experienced neuropathologists at each local site.

LB disease staging was rated according to the criteria of Braak, Del Tredici, Rüb, de Vos, Jansen Steur and Braak [14]. While not systematically reported, in some cases—particularly those unclassifiable according to the Braak LB system—the neuropathological report specified individual regions of LB pathology (neocortical, brain stem, amygdala, or limbic), which are reported here for additional context.

Presence of cerebrovascular findings were rated according to VCING criteria [15], assessing the individual presence or absence of subcortical infarcts > 10 mm, moderate/severe occipital leptomeningeal cerebral amyloid angiopathy (CAA), and moderate/severe occipital white matter (WM) arteriosclerosis.

Also assessed were presence/absence of limbic-predominant age-related TDP-43 encephalopathy neuropathological changes (LATE-NC) [16], frontotemporal lobar degeneration (FTLD) [17], corticobasal degeneration (CBD) [18], and argyrophilic grain disease (AGD) [19], while other less common brain diseases were also considered on a case-by-case basis.

#### Indices of multiple deprivation

Indices of multiple deprivation (IMD) were derived for each decedent’s home postcode. These were adjusted as described by Abel, Barclay and Payne [20] to allow for the accurate inclusion of cases from across the UK constituent nations, normalised to English indices. IMD ranks were divided at quintiles into five strata, with the first therefore including those from the 20% least deprived areas of England, and the fifth including those from the 20% most deprived areas (or the adjusted equivalent from the rest of the UK).

### Statistical analysis

Severity of staged neuropathological changes (Thal amyloid phase, Braak neurofibrillary tangle stage, CERAD neuritic plaque score, and Braak Lewy body stage) and presence/absence of other changes (any Lewy pathology, subcortical cerebral infarcts > 10 mm, moderate/severe occipital leptomeningeal CAA, moderate/severe occipital WM arteriosclerosis, LATE-NC) were assessed with ordinal and binary logistic models, respectively. Lewy body disease was assessed with both Braak LB staging and binary models due to well-documented limitations of the varying historical approaches to staging methods for LB disease, with the Braak LB staging in particular having high non-classifiability [21]. For this purpose, LB disease was treated as either simply absent or present (Braak LB stage ≥ 1 or any report of LB disease).

Brain bank site was included as a random effect in all models to account for non-generalisable differences in sampling and semi-quantitative assessments between sites. IMD was included as a discrete fixed effect, with the lowest deprivation group treated as the reference. All models controlled for the decedent’s age at death, whether they were a carrier of an APOE ε4 allele, sex, and self/informant-reported medical history of hypertension, diabetes, smoking, and alcohol misuse.

All analyses were conducted in *R* statistical software with the *ordinal* and *lme4* packages for ordinal and binary logistic models, respectively. Resulting confidence intervals are presented without adjustment for multiple comparisons.

## Results

### Distribution of deprivation strata across cohort and sites

Seven-hundred and eighty-nine decedents had provided sufficient information to determine their UK-adjusted IMD. Fifty-seven had postcodes which could not be matched to an IMD rank and so were excluded from subsequent analysis; these did not significantly differ from those with valid IMD ranks in their baseline age (Z = 1.55, *p* = 0.12), education level (Z = − 0.85, *p* = 0.40), or male/female proportion (χ(1)^2^ = 0.26, *p* = 0.61). Missing IMD data were most common at the Oxford site, occurring for 14.4% of cases, followed by Cardiff (7.1% missing), Newcastle (5.7%), London (4.0%), Bristol (0.9%), and Manchester (0.8%). Five-hundred and ninety-nine cases had undergone APOE genotyping. There was a mean interval of 0.9 years (SD = 0.9) between the final observation and date of death.

**Fig. 1:**
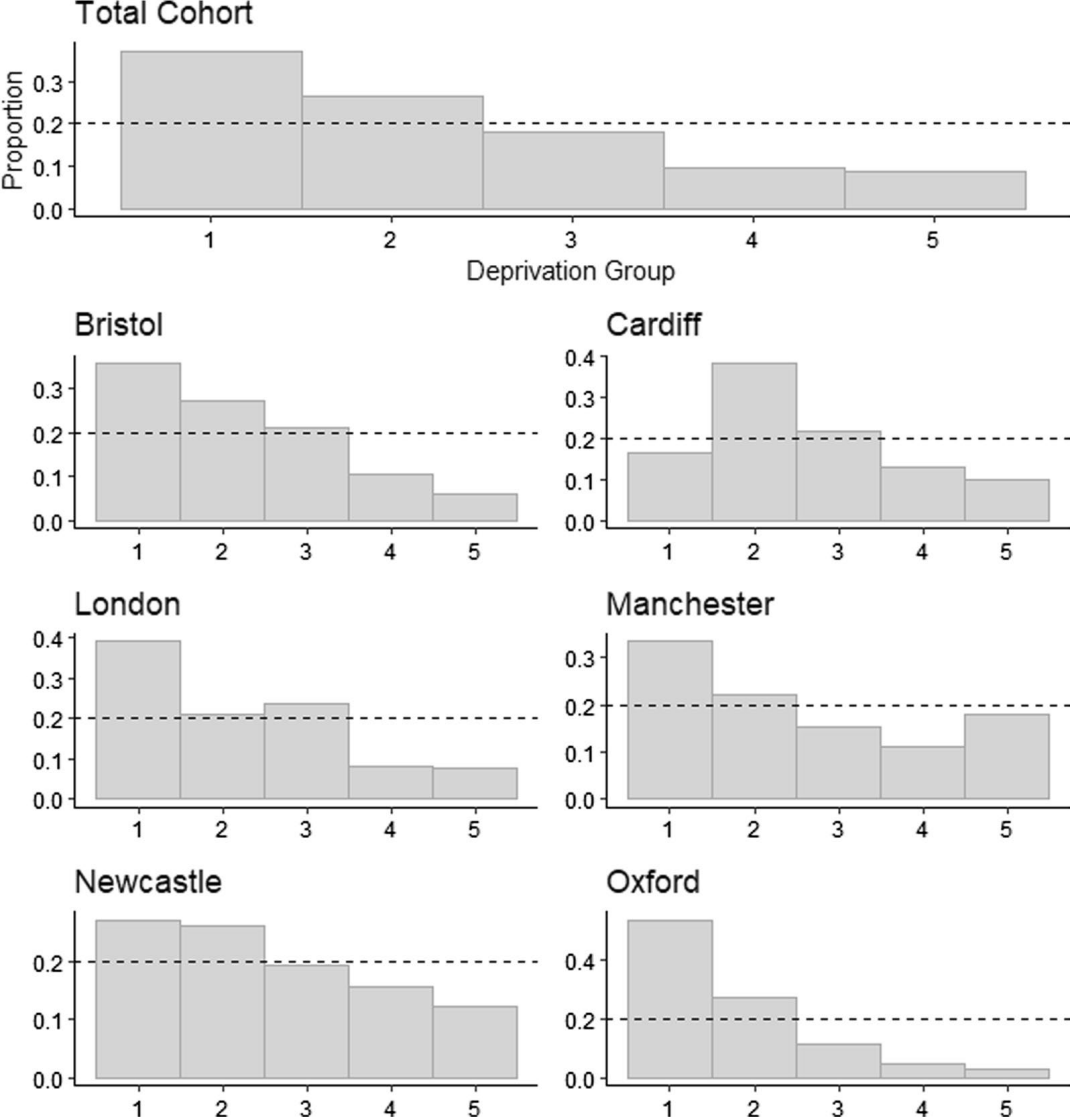
Proportional distributions of deprivation strata in the BDR cohort. Deprivation strata from least deprived (1) to most deprived (5). Dashed line indicates expected proportion if groups were to be uniformly distributed

Multiple deprivation quintile-groups, which due to their definition as percentile ranks should be uniformly distributed across the UK, were positively skewed in this sample (Fig. 1). Overall, and in all sampling sites excepting Cardiff, the lowest deprivation group were the most represented. Excepting Manchester, the most deprived areas were the least represented.

### Characteristics of deprivation strata

Clinical characteristics of each deprivation group are presented in Table 1, and neuropathological characteristics in Table 2. While overall cognitive impairments were not any more common in more deprived areas, in an ordinal logistic mixed model age- and APOE-adjusted CDR scores were significantly more severe in the fourth (Odds Ratio (95% CI) = 1.89 (1.08 – 3.34)) and fifth (Odds Ratio (95% CI) = 1.96 (1.06 – 3.62)) deprivation strata than in the first.

**Table 1:** Characteristics of the BDR cohort, stratified by deprivation level

	Deprivation group					
	1—Least Deprived	2	3	4	5—Most deprived	
	(N = 293)	(N = 209)	(N = 142)	(N = 77)	(N = 68)	*p* value
Age at death (years)	85.3 (8.43)	84.1 (8.60)	85.4 (8.60)	84.0 (8.25)	84.5 (9.36)	.61
Female sex	143 (48.8%)	98 (46.9%)	68 (47.9%)	32 (41.6%)	32 (47.1%)	.77
APOE ε4 non-carrier	97 (33.1%)	83 (39.7%)	51 (35.9%)	36 (46.8%)	21 (30.9%)	.29
APOE ε4 carrier	120 (41.0%)	77 (36.8%)	54 (38.0%)	25 (32.5%)	35 (51.5%)	
APOE genotype not known	76 (25.9%)	49 (23.4%)	37 (26.1%)	16 (20.8%)	12 (17.6%)	
Hypertension present	150 (51.2%)	106 (50.7%)	86 (60.6%)	32 (41.6%)	36 (52.9%)	.08
Diabetes present	27 (9.2%)	19 (9.1%)	23 (16.2%)	7 (9.1%)	13 (19.1%)	.15
Any cognitive impairment present	165 (56.3%)	125 (59.8%)	82 (57.7%)	48 (62.3%)	41 (60.3%)	.91

Mean (SD) or Count (%); p value from permutation test

**Table 2:** Incidence and rated severity of key neuropathological changes across area deprivation groups

	1—least deprived N = 293		2 N = 209	3 N = 142	4 N = 77	5—most deprived N = 68
Alzheimer’s Disease Neuropathological Change						
Thal Amyloid Phase	4 [1, 5]		3 [1, 5]	3 [1, 5]	3 [1, 4]	4 [2, 5]
Braak Tangle Stage	4 [2, 5]		3 [2, 6]	3 [2, 5]	3 [2, 5]	5 [3, 6]
CERAD Plaque Score	2 [0, 3]		2 [0, 3]	1 [0, 3]	1 [0, 3]	2 [1, 3]
Lewy Body Disease						
Any LB Disease	94 (32.1%)		63 (30.1%)	35 (24.6%)	19 (24.7%)	22 (32.4%)
Braak LB Stage	0 [0, 3]		0 [0, 4]	0 [0, 0]	0 [0, 1]	0 [0, 3]
Amygdala LBs	17 (5.8%)		8 (3.8%)	6 (4.2%)	2 (2.6%)	2 (2.9%)
Limbic LBs	13 (4.4%)		16 (7.7%)	8 (5.6%)	4 (5.2%)	6 (8.8%)
Brainstem LBs	6 (2.0%)		2 (1.0%)	4 (2.8%)	1 (1.3%)	0 (0%)
Neocortical LBs	32 (10.9%)		16 (7.7%)	8 (5.6%)	5 (6.5%)	5 (7.4%)
Cerebrovascular Disease						
Infarct(s) > 10 mm	33 (11.3%)		15 (7.2%)	13 (9.2%)	6 (7.8%)	9 (13.2%)
Moderate/severe CAA	86 (29.4%)		66 (31.6%)	38 (26.8%)	23 (29.9%)	27 (39.7%)
Moderate/Severe WM Arteriosclerosis	71 (24.2%)		40 (19.1%)	30 (21.1%)	11 (14.3%)	12 (17.6%)
TDP-43 Pathology						
LATE-NC	79 (27.0%)		45 (21.5%)	27 (19.0%)	16 (20.8%)	15 (22.1%)
FTLD-TDP	2 (0.6%)		4 (2.0%)	1 (0.7%)	3 (3.9%)	1 (1.5%)
Not Assessed	25 (8.5%)		6 (2.9%)	10 (7.0%)	1 (1.3%)	1 (1.5%)
Other Findings						
Any FTLD	8 (2.7%)		6 (2.9%)	5 (3.5%)	4 (5.2%)	4 (5.9%)
Corticobasal Degeneration	2 (0.7%)		2 (1.0%)	0 (0%)	0 (0%)	2 (2.9%)
Argyrophilic Grain Disease	6 (2.0%)		5 (2.4%)	2 (1.4%)	1 (1.3%)	1 (1.5%)

Median [Interquartile Range] or Count (%)

### Primary analysis: associations between area deprivation and common neuropathological findings

Mixed-effects logistic models were estimated for each major neuropathological finding: findings with notably low incidence (FTLD, CBD, AGD; see Table 2) were not modelled at this stage.

### Alzheimer’s disease neuropathologic change

Individuals from the most deprived areas of England or Wales were significantly more likely to have more severe Braak neurofibrillary tangle staging and CERAD plaque score than those from the least deprived areas (see Table 3 for adjusted odds). This association was not observed for Thal phase of amyloid deposition.

**Table 3:** Association between area deprivation and grading of Alzheimer’s disease neuropathologic changes

Adjusted Odds	Thal Amyloid Phase		Braak Tangle Stage		CERAD Plaque Score	
	Odds Ratios	CI	Odds Ratios	CI	Odds Ratios	CI
IMD Stratum 2 vs 1	1.26	0.77 – 2.06	1.23	0.79 – 1.90	1.26	0.77 – 2.05
IMD Stratum 3 vs 1	1.18	0.70 – 2.00	1.06	0.66 – 1.70	1.05	0.62 – 1.78
IMD Stratum 4 vs 1	0.76	0.42 – 1.39	0.81	0.45 – 1.46	0.72	0.38 – 1.38
IMD Stratum 5 vs 1	1.37	0.71 – 2.62	1.81	1.01 – 3.23	2.09	1.09 – 3.98
Covariates:						
Age at Death	1.01	0.99 – 1.03	0.97	0.95 – 0.99	0.99	0.97 – 1.01
APOE ε4 Carrier	3.78	2.57 – 5.56	3.38	2.38 – 4.80	3.19	2.17 – 4.67
Diabetes	0.71	0.39 – 1.29	0.83	0.48 – 1.43	0.86	0.47 – 1.56
Hypertension	0.84	0.58 – 1.21	0.70	0.50 – 0.98	0.69	0.48 – 1.00
Male Sex	0.98	0.67 – 1.44	0.81	0.57 – 1.15	0.84	0.57 – 1.24
Smoker	0.98	0.66 – 1.44	1.20	0.84 – 1.70	0.96	0.65 – 1.41
Alcohol Misuse	0.95	0.50 – 1.82	1.17	0.65 – 2.08	1.66	0.83 – 3.31

### Lewy body disease

There was no clear association between area deprivation and either the severity of Braak-staged LBD, or odds of any LBD being present, including non-Braak LB staged cases (see Table 4). There was also not any clear association between APOE4 allele presence and either the presence of LBD, or of higher severity of LBD.

**Table 4:** Association between area deprivation and Lewy body disease

Adjusted Odds	Braak Lewy Body Stage		Any Lewy Body Disease	
	Odds Ratios	CI	Odds Ratios	CI
IMD Stratum 2 vs 1	0.91	0.51 – 1.62	0.92	0.54 – 1.57
IMD Stratum 3 vs 1	0.60	0.30 – 1.19	0.59	0.31 – 1.12
IMD Stratum 4 vs 1	0.61	0.27 – 1.39	0.53	0.24 – 1.14
IMD Stratum 5 vs 1	1.20	0.61 – 2.37	0.97	0.49 – 1.93
Covariates:				
Age at Death	1.00	0.97 – 1.03	1.00	0.97 – 1.02
APOE ε4 Carrier	1.35	0.86 – 2.10	1.36	0.89 – 2.07
Diabetes	0.47	0.20 – 1.12	0.54	0.25 – 1.14
Hypertension	0.66	0.42 – 1.04	0.75	0.49 – 1.15
Male Sex	1.46	0.91 – 2.34	1.40	0.90 – 2.19
Smoker	1.18	0.74 – 1.89	1.21	0.77 – 1.89
Alcohol Misuse	1.06	0.49 – 2.29	1.10	0.54 – 2.25

### Cerebrovascular disease

Consistent with the associations observed in AD associated neuropathology, those from the most deprived areas had a significantly higher probability of featuring CAA at autopsy. However, there was no observed association between area deprivation and risk of either infarcts or WM arteriosclerosis being present (see Table 5).

**Table 5:** Association between area deprivation and hallmarks of cerebrovascular disease

Adjusted Odds	Subcortical Cerebral Infarct(s) > 10 mm		Moderate/Severe Occipital Leptomeningial Cerebral Amyloid Angiopathy		Moderate/Severe Occipital White Matter Arteriosclerosis	
	Odds Ratios	CI	Odds Ratios	CI	Odds Ratios	CI
IMD Stratum 2 vs 1	0.52	0.19 – 1.37	1.42	0.76 – 2.64	0.75	0.38 – 1.48
IMD Stratum 3 vs 1	1.08	0.41 – 2.86	0.86	0.42 – 1.79	1.32	0.64 – 2.71
IMD Stratum 4 vs 1	0.75	0.24 – 2.35	0.63	0.26 – 1.50	0.37	0.13 – 1.07
IMD Stratum 5 vs 1	1.33	0.50 – 3.56	2.55	1.18 – 5.53	1.09	0.47 – 2.52
Covariates:						
Age at Death	1.04	1.00 – 1.08	1.02	1.00 – 1.05	1.03	1.00 – 1.06
APOE ε4 Carrier	0.69	0.35 – 1.36	2.74	1.69 – 4.44	1.03	0.62 – 1.72
Diabetes	0.40	0.11 – 1.43	0.76	0.35 – 1.68	1.13	0.51 – 2.49
Hypertension	0.75	0.39 – 1.44	1.18	0.73 – 1.91	1.17	0.71 – 1.95
Male Sex	2.42	1.18 – 4.98	1.70	1.02 – 2.82	1.48	0.87 – 2.53
Smoker	1.67	0.84 – 3.32	0.83	0.50 – 1.39	0.75	0.43 – 1.30
Alcohol Misuse	0.43	0.09 – 1.99	1.47	0.59 – 3.67	1.86	0.73 – 4.76

### TDP-43 proteinopathy

There was no association between area deprivation and presence of LATE-NC (see Table 6).

**Table 6:** Association between area deprivation and TDP-43 neuropathological changes

Adjusted Odds	Limbic-Predominant Age-Related TDP-43 Encephalopathy	
	Odds Ratios	CI
IMD Stratum 2 vs 1	0.76	0.38 – 1.48
IMD Stratum 3 vs 1	0.77	0.35 – 1.67
IMD Stratum 4 vs 1	0.65	0.25 – 1.64
IMD Stratum 5 vs 1	0.84	0.36 – 1.95
Covariates:		
Age at Death	1.07	1.03 – 1.11
APOE ε4 Carrier	1.59	0.94 – 2.67
Diabetes	1.01	0.44 – 2.35
Hypertension	0.45	0.27 – 0.77
Male Sex	1.29	0.75 – 2.23
Smoker	0.96	0.56 – 1.64
Alcohol Misuse	0.57	0.19 – 1.66

### Exploratory analysis of additional neuropathological findings

We conducted an exploratory analysis of the presence of additional neuropathological findings, or specific patterns of pathology: FTLD, CBD, and AGD. Due to the limited number of cases for these we assessed only the unadjusted odds for each deprivation stratum.

In all cases, there was no clear association between area deprivation and the occurrence of these rarer neuropathological changes (see Table 7).

**Table 7:** Unadjusted associations between area deprivation and rare neuropathological changes

Unadjusted Odds	Any Frontotemporal Lobar Degeneration		Corticobasal Degeneration		Argyrophilic Grain Disease	
	Odds Ratios	CI	Odds Ratios	CI	Odds Ratios	CI
IMD Stratum 2 vs 1	0.90	0.31 – 2.66	1.37	0.19 – 9.81	1.14	0.34 – 3.80
IMD Stratum 3 vs 1	1.20	0.38 – 3.74	No Cases		0.68	0.14 – 3.43
IMD Stratum 4 vs 1	1.69	0.49 – 5.88	No Cases		0.62	0.07 – 5.19
IMD Stratum 5 vs 1	2.11	0.60 – 7.33	4.32	0.60 – 31.26	0.70	0.08 – 5.91

### Sensitivity analysis

We theorised that the observed effects could be otherwise explained if those from different deprivation strata were motivated to volunteer for tissue donation for different reasons: some may participate due to their own diagnosis of a cognitive impairment, while others may participate for other reasons (e.g. scientific interest). The former group would be expected to have greater incidence or severity of neuropathological changes; if this group were over-represented in more deprived sampling areas, then this could explain the associations found between deprivation and neuropathological changes.

We therefore conducted a sensitivity analysis conditioning on individuals’ rated cognitive impairment status on the CDR; a CDR score of ≥ 0.5 at baseline assessment was taken as evidence that cognitive impairment of any level was present (see Table 1), which could account for sampling bias.

Conditioning on the presence of any cognitive impairment at baseline did not meaningfully change the results of these models: there remained an association between residing in the most deprived areas and greater severity of NFT and neuritic plaque staging (though adjusting for this highly prognostic variable widened the confidence intervals in the former to include a possible null effect), as well as a higher prevalence of moderate/severe CAA (see Additional file 1: Table s1).

In a further sensitivity analysis adjusted instead by a CDR of ≥ 1, rather than 0.5, the associations between area deprivation and neuropathological changes were no longer observed for Braak tangle stage (Odds Ratio (95% CI) = 1.51 (0.71 – 3.22)) or CERAD score (Mixed model not estimable: fixed effects Odds Ratio (95% CI) = 1.65 (0.86 – 3.16)), but the association remained with presence of CAA (Odds Ratio (95% CI) = 3.81 (1.44 – 10.08)).

## Discussion

Dementia is known to be more common amongst more disadvantaged communities and neighbourhoods [22]. We aimed to assess whether area deprivation contributes to these inequalities through an association with different levels of neuropathological change at autopsy in UK brain banks. We found that those living within the top 20% most deprived areas of England and Wales were at greater risk of more severe AD-related neuropathological changes, with higher severity of NFT and neuritic plaque staging, and greater risk of CAA. This was the case after adjusting for theorised health factors which might mediate this (e.g. smoking and diabetes), as well as for non-generalisable regional differences. These results suggest that, in addition to associations with reduced cognitive reserve, and barriers to diagnosis and treatment, area deprivation may therefore also contribute to dementia-related health inequalities through a greater severity of AD-related neuropathological changes in those with cognitive impairments.

This builds on previous research which observed an increasing prevalence of pathological changes meeting criteria for diagnosis of AD with increasing area deprivation in the US [8], showing that this is particularly driven by an increase in the severity of both NFT and neuritic plaques. We also show that there is a greater risk of CAA. Finally, we demonstrate that these findings remain after adjusting for APOE genotype, as well as recognised health and lifestyle factors (diabetes, hypertension, smoking, and alcohol misuse). However, in contrast to previous findings, we did not observe a linear dose-dependent effect of increasing deprivation: rather we observed only that those from the most deprived area group were at risk of greater disease severity, with intermediate groups showing no significant difference from the least deprived group.

A number of unassessed mediating factors could explain this association; for example, peripheral inflammation and hypothalamic–pituitary–adrenal (HPA) axis dysregulation have been mooted as possible mediators between earlier-life disadvantage and adverse experiences, and the genesis of a range of mid- and later-life diseases [23]. Peripheral inflammation and HPA axis dysregulation have been specifically linked to AD [24], and the former may be higher [25] and progressively increasing [26] at the prodromal stages of clinical AD (and dementia with Lewy bodies), suggesting an early association in the emergence of the clinical syndrome – possibly through greater vulnerability to formation of neuritic plaques, NFTs, and CAA. However, without any objective measurements of HPA axis dysregulation, chronic stress, or inflammation, we are unable to test whether these mediate the observed associations.

There was no greater risk associated with area deprivation observed for either presence or severity of LB disease, other CVD findings, or LATE-NC. This could reflect fundamental differences in the pathogenesis of these aetiologies, or a lack of power to detect any meaningful differences due to the lower prevalence of non-AD changes in some cases. We also found no association between area deprivation and rarer pathologies (FTLD, CBD, AGD).

There was also no association between area deprivation and Thal phase of amyloid deposition, despite an association between area deprivation and another measure of amyloid pathology (CERAD scoring of neuritic amyloid plaques). While Thal phase was generally high in all sub-groups, recent evidence has suggested that this has substantially less association with cognitive outcomes than do CERAD score and Braak NFT staging [27], and consequently CERAD score and Thal phase may not be equivalent measures.

We theorised that the effect in AD pathology could be otherwise explained in whole or in part if those from different areas volunteer for brain tissue donation for different reasons: e.g., if people from less deprived backgrounds are more likely to donate brain tissue as a healthy control without cognitive impairment, while those from more deprived backgrounds may be more likely to volunteer for brain tissue as a cognitively impaired case. We found mixed support for this in two sensitivity analyses: the association between area deprivation and NFT staging was no longer significant after conditioning on baseline cognitive impairment (this widened the confidence intervals, but did not meaningfully changing the point estimate), but the associations between deprivation and both neuritic plaque staging and presence of CAA remained significant. However, when excluding mildly impaired cases from the cognitively impaired group, there was only an apparent association between area deprivation and CAA.

Therefore, while differences in the proportion of cases and controls across deprivation strata did not appear to account for the observed relationship with neuropathological changes, cognitive impairments, when present, were typically more severe in those from the most deprived areas. This might partially account for the associations seen between deprivation and some neuropathological changes, however, the causal direction is unclear; sampling of more cognitively impaired cases from more deprived areas could lead to the selection of cases with more severe neuropathological changes. Conversely, those from more deprived areas may have a greater severity of cognitive impairment as a result of their greater severity of neuropathological change.

### Limitations

These findings suggest that health inequalities relating to dementia may go beyond the diagnostic and therapeutic biases or deprivation-related health factors previously described; socioeconomic disadvantage may be associated with greater neuropathological change, and this may not be fully explained by mooted deprivation-associated health behaviours (e.g. smoking) or comorbidities (e.g. diabetes or hypertension). Prevention of dementia may therefore require a better understanding of the role of lifetime disadvantage in the emergence and severity of neuropathological changes. Other potential mediators, such as chronic stress and systemic- or neuro-inflammation [23] which we could not assess here, might account for the relationship and other studies need to address such limitations.

We describe relative risks of specific neuropathological changes, however, we cannot infer the national prevalence of these neuropathological changes meaningfully from these data. The majority of cases in this cohort have some degree of cognitive impairment, and there is an under-representation of more disadvantaged areas relative to the target population. By weighting observations according to these, and other important characteristics, future research may provide better evidence of the prevalence of these neuropathological changes, rather than relative risks alone.

These results demonstrate the importance of increasing the engagement of those from more deprived backgrounds in neurodegenerative disease research. There was a notable bias, consistent across sites, towards sampling from less deprived areas of the United Kingdom, and those with cognitive impairments in these areas were typically more impaired. Associations between community deprivation and research non-participation are well-recognised, and inequalities in non-participation are increasing [28]. This remains a limitation of this work and should be a priority to address in future research into dementia and neuropathological changes.

## Conclusions

Those in the most deprived areas of the United Kingdom have a greater severity of AD-related neuropathological changes: Braak NFT staging, CERAD plaque scores, and CAA. This was not explained by APOE genotype, smoking or alcohol use, or cardiovascular risk factors.

## Supplementary Information

**Additional file 1**. Supplementary Table S1. Logistic models adjusting for baseline cognitive status.

## Data Availability

All data supporting these analyses are available through the MRC Dementias Platform UK (DPUK) and the UK Brain Bank Network (UKBBN).

## Abbreviations

AD: Alzheimer’s disease
AGD: Argyrophilic grain disease
APOE: Apolipoprotein E
BDR: Brains for Dementia Research
CAA: Cerebral amyloid angiopathy
CAMDEX: Cambridge Examination for Mental Disorders of the Elderly – Medical History
CBD: Corticobasal degeneration
CDR: Clinical dementia rating
CVD: Cerebrovascular disease
CERAD: Consortium to Establish a Registry for Alzheimer's Disease
DPUK: Dementias platform UK
FTLD: Fronto-temporal lobar degeneration
IMD: Indices of multiple deprivation
LATE-NC: Limbic-predominant age-related TDP-43 encephalopathy neuropathological changes
LB: Lewy body
MRC: The Medical Research Council
NFT: Neurofibrillary tangle
VCING: Vascular cognitive impairment neuropathology guidelines
WM: White matter
