# Supplementary material for: Neurodegenerative brain changes are associated with area deprivation in the United Kingdom: findings from the Brains for Dementia Research study
Source: Acta Neuropathol Commun. 2021 Dec 19;9:198. doi: 10.1186/s40478-021-01301-8 (PMC8684681; doi:10.1186/s40478-021-01301-8)
Supplement: Supplementary file 1 — Additional file 1. Supplementary Table S1. Logistic models adjusting for baseline cognitive status. [file 40478_2021_1301_MOESM1_ESM.docx]

**Supplementary Table S1. Logistic models adjusting for baseline cognitive status.**

|  | **Braak Tangle Stage** | | **CERAD Plaque Score** | | **Cerebral Amyloid Angiopathy** | |
| --- | --- | --- | --- | --- | --- | --- |
| ***Adjusted Odds*** | *Odds Ratios* | *CI* | *Odds Ratios* | *CI* | *Odds Ratios* | *CI* |
| *IMD Stratum 2 vs 1* | 1.22 | 0.82 – 1.81 | 1.20 | 0.78 – 1.86 | 1.44 | 0.85 – 2.43 |
| *IMD Stratum 3 vs 1* | 0.77 | 0.49 – 1.19 | 0.85 | 0.52 – 1.39 | 1.14 | 0.61 – 2.13 |
| *IMD Stratum 4 vs 1* | 0.67 | 0.38 – 1.19 | 0.70 | 0.38 – 1.30 | 0.92 | 0.43 – 1.96 |
| *IMD Stratum 5 vs 1* | 1.79 | 0.99 – 3.22 | 1.98 | 1.02 – 3.86 | 2.31 | 1.09 – 4.88 |
| **Covariates:** |  |  |  |  |  |  |
| *Age at Death* | 0.99 | 0.97 – 1.01 | 1.00 | 0.98 – 1.02 | 1.02 | 1.00 – 1.04 |
| *APOE ε4 Carrier* | 2.22 | 1.60 – 3.09 | 2.50 | 1.74 – 3.58 | 2.15 | 1.40 – 3.32 |
| *Cognitive Impairment Present* | 7.31 | 4.98 – 10.72 | 5.82 | 3.92 – 8.63 | 2.19 | 1.31 – 3.65 |
|  |  | |  | |  | |
